# Precipitation Complexity and its Spatial Difference in the Taihu Lake Basin, China

**DOI:** 10.3390/e21010048

**Published:** 2019-01-10

**Authors:** Jian Hu, Yong Liu, Yan-Fang Sang

**Affiliations:** 1 Key Laboratory of Water Cycle and Related Land Surface Processes, Institute of Geographic Sciences and Natural Resources Research, Chinese Academy of Sciences, Beijing 100101, China; 2 State Key Laboratory of Hydrology-Water Resources and Hydraulic Engineering, Nanjing Hydraulic Research Institute, Nanjing 210029, China

**Keywords:** precipitation, complexity, sample Entropy, Taihu Lake Basin, urbanization

## Abstract

Due to the rapid urbanization development, the precipitation variability in the Taihu Lake basin (TLB) in East China has become highly complex over the last decades. However, there is limited understanding of the spatiotemporal variability of precipitation complexity and its relationship with the urbanization development in the region. In this article, by considering the whole urbanization process, we use the SampEn index to investigate the precipitation complexity and its spatial differences in different urbanization areas (old urban area, new urban area and suburbs) in TLB. Results indicate that the precipitation complexity and its changes accord well with the urbanization development process in TLB. Higher urbanization degrees correspond to greater complexity degrees of precipitation. Precipitation in old urban areas shows the greatest complexity compared with that in new urban areas and suburbs, not only for the entire precipitation process but also the precipitation extremes. There is a significant negative correlation between the annual precipitation and its SampEn value, and the same change of precipitation can cause a greater complexity change in old urbanization areas compared with the new urban areas and suburbs. It is noted that the enhanced precipitation complexity in a new urban area during recent decades cannot be ignored facing the expanding urbanization.

## 1. Introduction

Under the aggravating influence of climate change and anthropogenic effects, the hydrological cycles in many basins and regions worldwide are significantly changing [1,2]. As an important part of the hydrological cycle, precipitation and its variability and change are often related to water disasters. They can cause adverse consequences to economic development and people’s lives in affected regions [3,4]. Therefore, detecting the precipitation variability under the changing environment is important and is a necessary basis for better understanding hydrological variability, effectively implementing the prevention of flood-waterlog and drought disasters, and planning and managing water resources.

Hydrological time series is the embodiment and reflection of the comprehensive effects of hydrological variability [5,6] with complex evolution at multi-time scales [7]. Thus, hydrological time series analysis is an effective approach to identify the hydrological variability and changes and further separates the different components in a hydrological time series by considering statistical significance [8,9]. Traditional methods are mainly based on probabilistic statistical theory to analyze certain variability types of hydrological time series, such as monotonic trend [10], jump (or phase trend) [11], periodicities [12], long-term dependence (correlation) [13], etc. However, they fail in identifying more complex hydrological variability [14].

Among those new theories for time series analysis, the information entropy theory is effective in quantifying the irregularity and complexity of dynamic systems [15,16]. Generally, more irregularity indicates more complex systems. It provides a novel approach to explore the complexity of hydrology processes [16,17,18,19,20,21,22]. Pincus [23,24] proposed the concept of approximate entropy to measure the complexity of nonlinear time series, but it has difficulty in calculation [25]. To overcome the problem, Richman and Moorman [26] proposed the sample entropy (SampEn) by avoiding count self-matches. SampEn has many advantages including non-dependence on data length, high consistency, insensitivity to missing data, and high computational efficiency, etc. [27]. It has been widely used in the various fields [28,29,30,31]. In Hydrology, the SampEn index is often used to analyze the complexity of hydrological variables [3,32,33,34]. It has been proved with better performance in calculating the complexity of precipitation and runoff at different timescales, compared with the approximate entropy [31,34].

Located in the Yangtze River Delta, the Taihu Lake Basin (TLB) is one of the most developed, densely populated and highly urbanized areas in China. The rapid urbanization process of TLB has a profound impact on the precipitation variability over recent decades. Many studies have investigated the precipitation variability and its physical causes in the region [35,36,37,38,39,40,41,42]. It is considered that there is a certain genetic correlation between the El Nino phenomenon and the precipitation change in TLB. [36]. Further, it is suggested that the rapid urbanization has great influence on the precipitation variability [37]. Higher urbanization level corresponds to a larger decrease of relative humidity and slower increase in precipitation due to the “rain island effect” [38]. Yang et al. [39] found that the rapid urbanization in recent years had a certain correlation with the increase of extreme precipitation in the Meiyu period (May to July) in TLB. Overall, these studies mainly use traditional statistical methods to investigate the trends and periodicities of precipitation at various characteristic scales. However, the total precipitation complexity and its spatial difference in TLB still lacs an in-depth study, which is an important basis for and prediction at large timescales.

By considering the whole urbanization process in TLB, the main objective of this study is to analyze the spatial and temporal complexity of precipitation in different urbanized areas in TLB by using the SampEn index and moving sample entropy (M-SampEn) index. Two open questions are to be answered: (1) How precipitation complexity changes in TLB and if there is spatial difference? and (2) how precipitation complexity responses to the urbanization in TLB?

## 2. Data and Methods

### 2.1. Study Area

**Figure 1:**
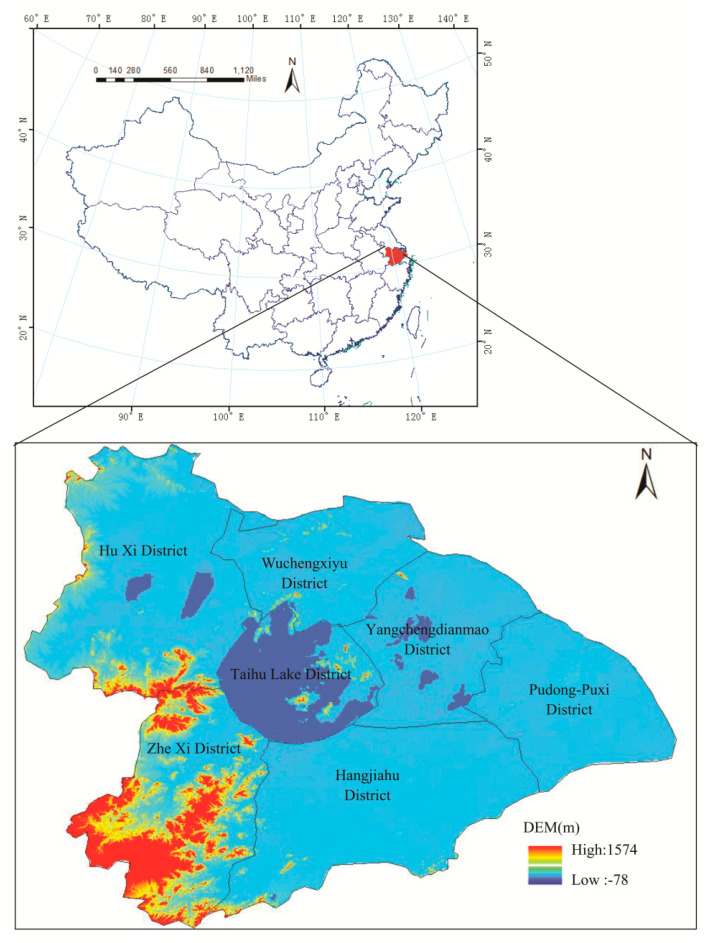
Location of the Taihu Lake Basin in China and its topography and seven sub-basins.

The TLB is located in the downstream tributary of the Yangtze River Basin in China. With a total area of 36895 km^2^, the basin is dominated by the plain, accounting for 4/6 of the total area, while the mountain and hills make up only 1/6 in the west, and the remaining 1/6 is composed of the interconnected and crisscrossed water systems. The topographic features in TLB are high in the surrounding area and low in the middle region. According to the terrain and water system of the watershed, the whole basin can be divided into seven parts, as shown in Figure 1.

The TLB has a typical subtropical monsoon climate, dry and cold in winter, but hot and humid in summer. The average annual precipitation in TLB is about 1130.8 mm (1965–2013), and the average rainfall in flood season (including rainy season from May to July, and typhoon season in August and September) is about 683.2 mm, accounting for 60.4% of annual precipitation.

**Figure 2:**
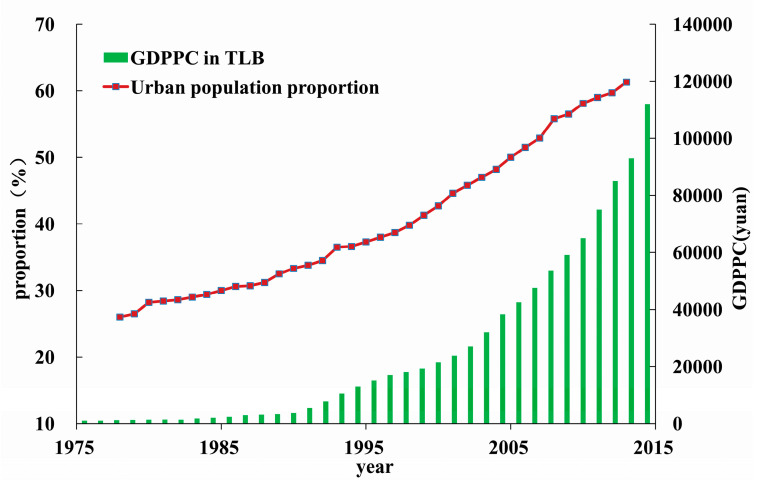
Population increase and economic development in the Taihu Lake Basin over the last four decades. GDPPC means the Gross Domestic Product per capita.

With a 77.6% urbanization rate in 2013, the TLB is one of the most urbanized areas in China. In 2013, the population in the basin was 59.97 million, and the Gross Domestic Product (GDP) reached ¥6.69 trillion, accounting for 9.9% of the national GDP, while the per capita GDP (GDPPC) is 2.3 times that of the whole country. Over the past decades, the large-scale expansion of city groups (centered on Shanghai, including Changzhou, Wuxi, Suzhou, Huzhou, Hangzhou, Jiaxing, and many rapidly developing towns) has been accompanied by the rapid succession of underlying surface and rapid accumulation of population and industry, which has been obviously changing the hydrological regimes in the region. The urbanization process in TLB has been rapidly increasing since the early 1990s. Figure 2 shows the increasing process of two urbanization indicators in TLB. The proportion of urban population in 2013 was 61.3%, with an average increase rate of 1.39% per year over the past decade, much bigger than 0.61% per year before the 1990s. The other indicator of GDPPC also indicates a similar phenomenon.

### 2.2. Data Used

The precipitation data measured at 49 weather stations in TLB, with the period of 1965–2013, are used for the study. The monthly precipitation data are used to interpret the spatial difference of precipitation complexity in TLB, and the daily precipitation data are used for analyzing the temporal variability of precipitation complexity. The quality, consistency and reliability of all data are checked to ensure the accuracy of the results. Due to the spatial heterogeneity in urbanization development, the precipitation complexity in TLB has a spatial difference. The effect of urbanization on precipitation changes is considered here by detecting the spatial division following urbanization, and the precipitation complexity in different urbanization areas is compared; after that, the relationship between precipitation complexity and urbanization development is explored.

**Figure 3:**
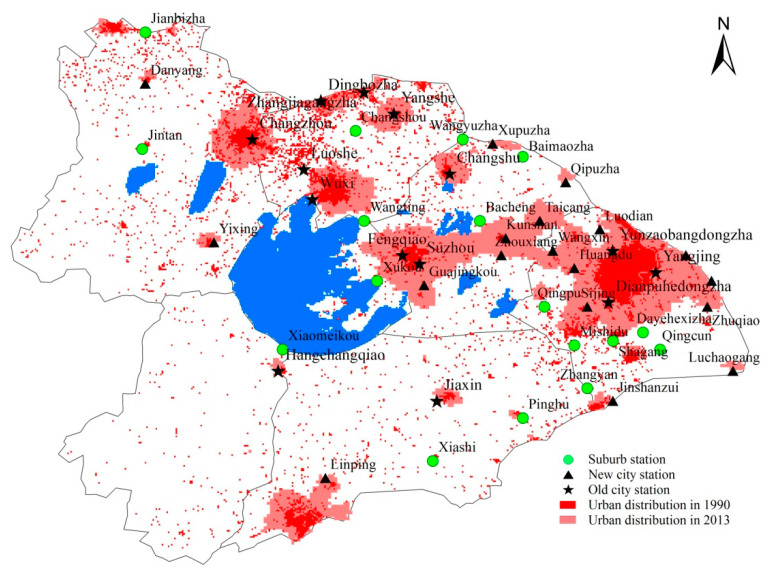
Spatial distribution of 49 weather stations in the Taihu Lake Basin. It includes 14 old city stations, 18 new city stations and 17 suburb stations.

In order to clarify the urbanization development and its impacts on the precipitation complexity in TLB, it is necessary to first analyze the temporal and spatial evolution of the urbanization process. For such a rapidly developing region, the areas with mature urbanization before 1990 are taken as old urban areas, while the fast-urbanizing areas after 1990 are taken as new urban areas, and the rest of the slow-urbanizing areas are suburbs. We calculate the built-up area ratios (the ratio of urban area in the total areas) around 5 km × 5 km of each station according to the urban distribution in 1990 and 2013. They are interpreted from the remote sensing images by using the nine-point classification method. According to the spatial distribution of built-up areas in 2013, those stations with built-up area ratios less than 66.7% (i.e., less than 6 points in the 9 points around 5 km × 5 km of the station) are considered as suburb stations, and the rest of the stations are further divided into old and new city stations based on the results in 1990, that is, the old city stations with the built-up area ratios greater than 66.7% but the new city stations less than 66.7% in 1990. It is known that new city stations are affected slightly by urbanization before 1990 but strengthened after 1990. The classification of stations is shown in Figure 3, including 14 old city stations, 18 new city stations and 17 suburb stations. The area precipitation data in the three areas are obtained by computing the average precipitation at the corresponding stations.

### 2.3. Methods 

The index of SampEn is used to quantify the complexity of precipitation in TLB. It is calculated as follows: 

(I) For a *N*-point precipitation series *x*(*i*) (1 < *i* < *N*), construct *N* − *m* + 1 new series *X*(*i*) of *m* dimensional vectors from series *x*(*i*):

(1) X(i)=[x(i),x(i+1),⋯x(i+m−1)],i=1∼(N−m+1)

(II) Define the maximum distance between corresponding scalars *x*(*i* + *k*) and *x*(*j* + *k*) as the distance d[X(i),X(j)] between vectors *X*(*i*) and *X*(*j*):

(2) $$\underset{j\neq i}{d}\left[X(i),X(j)\right]=\underset{k=0,\cdots ,m-1}{\max}(\left|x(i+k)-x(j+k)\right|)$$

(III) Set a threshold *r*, which is a preset tolerance factor and adopted as a percentage of series’ standard deviation, to measure the tolerance of mismatch between the two vectors *X*(*i*) and *X*(*j*). The two vectors *X*(*i*) and *X*(*j*) are considered as similar if *d*[*X*(*i*), *X*(*j*)] < *r*. The number of vectors *X*(*j*) (*j* ≠ *i*) being similar to *X*(*i*) is given as *B_i_*, and then the ratio of *B_i_* to the total number *N* − *m* − 1 is noted as *B_i_^m^*(*r*), and *B^m^*(*r*) is the average *B_i_^m^*(*r*) for all *i*:

(3) $$B^{m}(r)=\frac{1}{N-m}\sum_{i=1}^{N-m}B_{i}^{m}(r)=\frac{1}{N-m}\sum_{i=1}^{N-m}\frac{1}{N-m-1}B_{i}$$

(IV) Repeat steps (I)–(III) to yield *B^m^*^+1^(*r*) for vectors of length *m* + 1, and then the theoretical SampEn value of the series *x*(*i*) is given by:

(4) $$SampEn\left(m,r,N\right)=\underset{N\to \infty }{\lim}\left\{-\ln\left[\frac{B^{m+1}(r)}{B^{m}(r)}\right]\right\}$$

**Figure 4:**
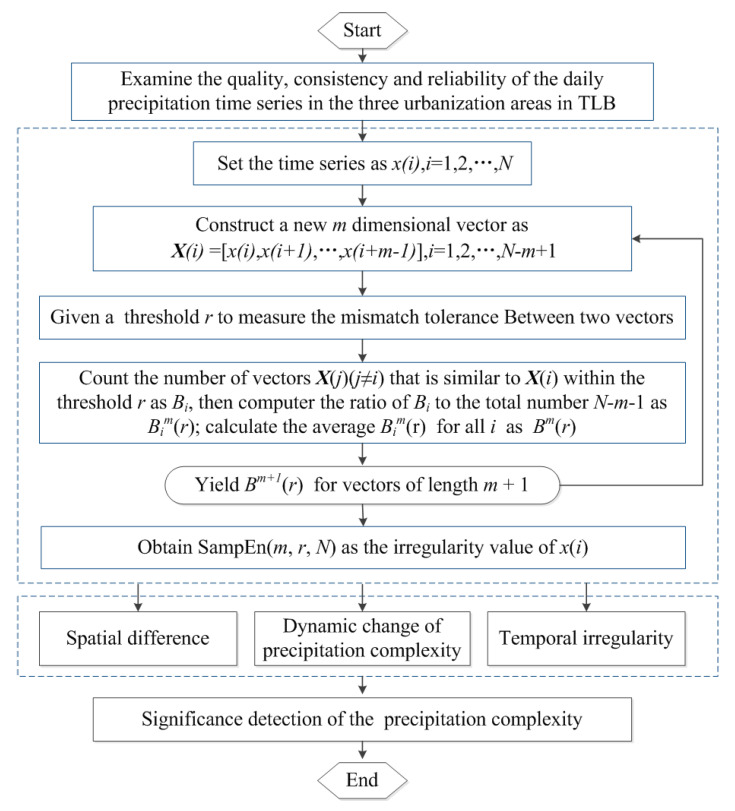
Flowchart of the sample entropy (SampEn) calculation used for the complexity analysis of precipitation in the Taihu Lake Basin.

In Equations (2)–(4), it is noted that j≠i, meaning that self-matches (compare a vector with itself) are excluded. The sample entropy for a finite length sequence can be simplified as:

(5) $$SampEn\left(m,r,N\right)=-\ln\left[\frac{B^{m+1}(r)}{B^{m}(r)}\right]$$

where *m* and *r* are two important parameters for computing the sample entropy. They would influence the sample entropy value but would not affect its variation due to the intrinsic consistency of sample entropy. Here, set *m* = 2 and *r* = 0.15*STD*, *STD* is the standard deviation of *x*(*i*), in the analysis of precipitation data in TLB. More details of the SampEn calculation can be found in Reference [26]. The analysis of precipitation complexity using the sample entropy index can also be found in Figure 4. Besides, the Mann-Kendall (MK) test is used to identify the significance of the precipitation and SampEn time series.

## 3. Results and Discussion

### 3.1. Spatial Difference of Precipitation Complexity in TLB

To analyze the spatial difference of precipitation complexity in TLB, the static SampEn values of the monthly precipitation data (with 588 samples) in the old urban area, new urban area and suburbs during 1965–2013 are calculated (shown in Table 1). It shows that the static SampEn values in the three areas have relative difference, with the highest value (2.168) in old urban areas, followed by new urban areas (2.103) and suburbs (2.066), reflecting the relative difference of precipitation complexity among them. Thus, it is thought that the spatial heterogeneity of urbanization development in TLB and its influence on the precipitation variability have spatial differences. Higher degree of urbanization corresponds to greater entropy value, greater precipitation complexity and lower predictability of precipitation.

### 3.2. Dynamic Change of Precipitation Complexity in TLB

**Figure 5:**
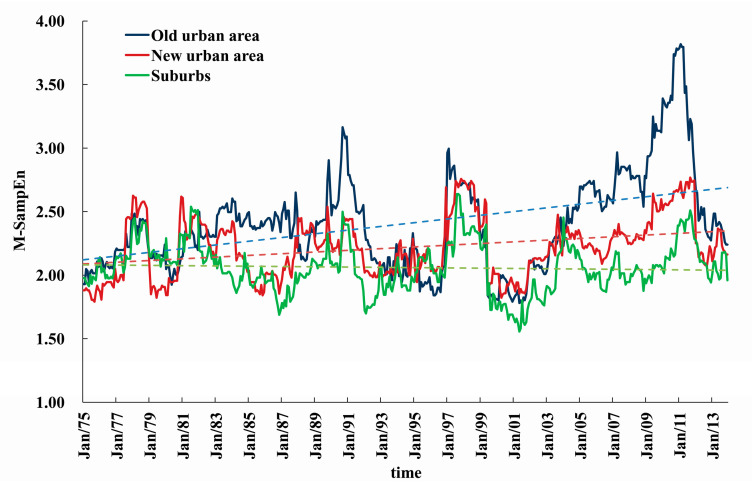
Variation of the moving SampEn (M-SampEn) value of the 120-month-length precipitation data in different urbanization zones in the Taihu Lake Basin. The x-coordinate represents the monthly time, for example, Jan/75 represents January in 1975, and the corresponding value is the SampEn value of the past 120 months precipitation series.

**Figure 6:**
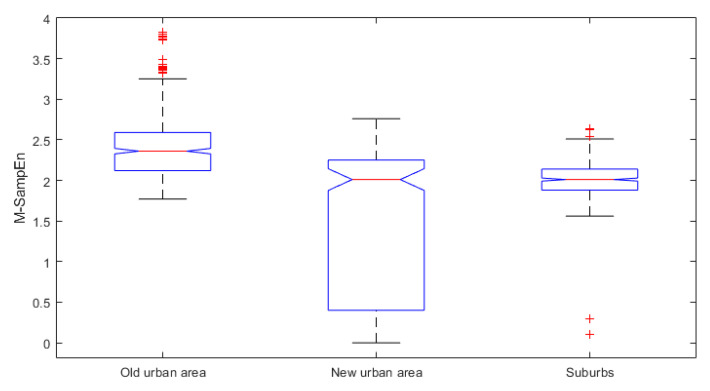
The box plots of the moving SampEn (M-SampEn) values of monthly precipitation data in the three urbanization areas of TLB.

The SampEn values can reflect the complexity of a time series, but it cannot dynamically quantify the change of its complexity over time. Here, the M-SampEn values of precipitation time series during 1965–2013 in the three areas are calculated to further depict the dynamic changes of precipitation complexity, where the 120 months is taken as the sliding window and 1 month as the slide step. The results are shown in Figure 5. In the whole period of 1965–2013, the M-SampEn values of monthly precipitation in the three urbanization areas of TLB are significantly different (*p* < 0.05), based on the one-way Analysis of Variance (ANOVA) at the 5% significance level (Table 2) and the box plots of M-SampEn values in Figure 6.

Furthermore, results in Figure 5 indicate that the precipitation complexity has periodic variability and increases with time, especially in the old urban area and new urban area. Before 1980, the M-SampEn values in the three areas are similar, but the difference gradually arises afterwards, especially for the extreme points. After 1980s, the SampEn value of precipitation in old urban areas shows greater complexity than that in new urban areas and suburbs. Particularly, the M-SampEn value in new urban areas also obviously increases after the 1990s, implying that the complexity of precipitation is enhanced by the rapid urbanization.

Meteorological and geographical conditions are the two main physical factors that influence regional precipitation. Because the city group in TLB is mainly located in the plain river network area, the difference of regional terrain conditions is less influential. Besides, the trends of annual precipitation in the old and new urban areas and suburbs are basically the same, and their correlation coefficient values are bigger than 0.88, which indicates that precipitation in different zones in TLB is controlled by the same climate conditions. However, there is a slight difference of M-SampEn values among the three zones, which is mainly attributed to the different urbanization degrees. Since 1990, the urbanization process in TLB has been accelerating, accompanied by the underlying surface succession, population growth and industrial development. All kinds of anthropogenic heat and carbon sources affect the physical and chemical properties of atmosphere. Therefore, the urbanization would inevitably cause the changes in local terrestrial surface and energy balance, which may cause mutual enhancement or superposition of urban "rain island effect" and "heat island effect", and increase precipitation complexity, especially in old urban areas and new urban areas that usually lead to large water accumulation and terrible urban waterlog problems.

### 3.3. Temporal Complexity of Precipitation in TLB

**Figure 7:**
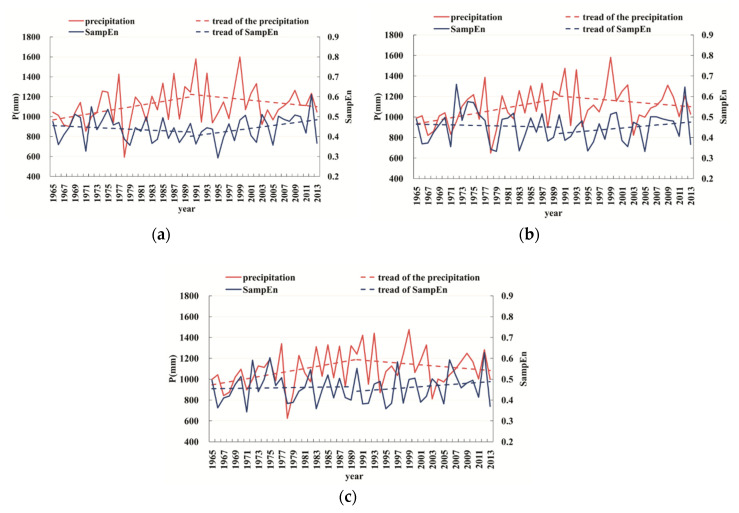
Annual precipitation and its corresponding SampEn value in the old urban areas (**a**), new urban areas (**b**) and suburbs (**c**) in the Taihu Lake Basin.

The SampEn value of daily precipitation data in each urbanization zone is calculated to further compare its difference with the annual precipitation in each year. As shown in Table 3, the annual precipitation shows a significant upward trend at 10% significance level before 1990, but decreases in all areas after 1990 (shown in Figure 7), although the trend is not significant. Furthermore, Figure 7 exhibits that there is a significant negative correlation between the long-term variation of annual precipitation and that of its SampEn value (with the negative correlation coefficient smaller than −0.30) and a good correlation between the peak precipitation and SampEn valley value. Larger annual precipitation corresponds to lower SampEn value and less complexity. Conversely, smaller annual precipitation with a bigger SampEn value is more complex.

We further calculate the ratio *u* between the relative changes in SampEn value and precipitation changes before and after 1990:

(6) $$u=\left|\frac{\rho_{SampEn,1991-2013}-\rho_{SampEn,1965-1990}}{\rho_{P,1991-2013}-\rho_{p,1965-1990}}\right|$$

where $\rho_{P}$ ($\rho_{SampEn}$) is the average change rate of annual precipitation (SampEn value). Table 4 shows that the value of *u* in old urban area is 0.00033, which is larger than 0.00021 in the new urban area and 0.00011 in suburbs. The *u* value in the old urban area is three times that in suburbs. It indicates that the impact of urbanization also varies with zones, which causes more precipitation decrease in the old urban area than that in new urban area, followed by suburbs. Therefore, the same precipitation change can cause greater change in precipitation complexity in the old urban area, although the new urban area is also greatly affected by urbanization.

## 4. Conclusions

The spatial heterogeneity of urbanization in TLB has a big influence on the spatial distribution of precipitation and its complexity in the region. In this study, the SampEn index is employed to investigate the spatial and temporal variability of precipitation complexity in different urbanization areas in TLB. Results indicated that the static SampEn value of precipitation in the old urban area is the highest, followed by the new urban area and suburbs, being consistent with the diverse development degrees of urbanization. Overall, a higher degree of urbanization causes a greater SampEn value of precipitation, higher precipitation complexity and lower predictability of precipitation.

Comparatively, precipitation in the old urban area shows more significant variability than that in the new urban area and suburbs, especially for extreme values, which involve the dynamic change of precipitation complexity. The precipitation complexity in new urban areas is enhanced by the rapid urbanization after 1990s. The complexity of precipitation in both old and new urban areas shows an upward trend, especially in recent years. The scale of urban agglomeration has been continuously expanding, and the urban “heat island effect” and “rain island effect” are intensified and superimposed on each other, causing the increase in urban precipitation complexity.

In the whole period, there is a significant negative correlation between annual precipitation and its SampEn value. Larger precipitation has a lower SampEn value and low complexity. By comparing the three urbanization zones, the relative complexity of precipitation in old urban area is the highest, indicating that the same precipitation changes can cause a greater change of precipitation complexity in the old urban area rather than the new urban area and suburbs.

In summary, the urbanization development significantly affects the spatial and temporal distribution of precipitation complexity in TLB, which will be aggravated along with urban agglomeration. It not only promotes us to further explore the physical mechanisms of precipitation change, but also provides a more useful guide for flood control and disaster mitigation, water resources management and project construction in TLB.

## Figures and Tables

**Table 1:** Static SampEn values of monthly precipitation data during 1965–2013 in different urbanization zones in the Taihu Lake Basin.

Urbanization Zone	Old Urban Area	New Urban Area	Suburbs
$\bar{P}$ * (mm)	1118.1	1100.1	1097.5
SampEn value	2.168	2.103	2.066

^*^$\bar{P}$ is the average annual precipitation.

**Table 2:** One-way Analysis of Variance (ANOVA) test results of the moving SampEn (M-SampEn) values of monthly precipitation data in the three urbanization areas of TLB at 5% significance level.

Source	Variance Sum	Degree of Freedom	Mean Value of Variance	Statistic Value *F*	Prob > *F*
Columns	154.822	2	77.411	171.61	2.170 × 10^−67^
Error	631.953	1401	0.451		
Total	786.775	1403			

**Table 3:** Trends of precipitation before and after 1990 in different urbanization areas in the Taihu Lake Basin.

Urbanization Zone		Z	$Z_{1-\alpha /2}$	Trend
Old urban area	Before 1990	1.94	1.64	significant upward
	After 1990	−0.12	1.64	insignificant
New urban area	Before 1990	2.42	1.64	significant upward
	After 1990	−0.42	1.64	insignificant
Suburbs	Before 1990	2.07	1.64	significant upward
	After 1990	−0.47	1.64	insignificant

**Table 4:** Difference of precipitation and SampEn values before and after 1990 in different urbanization zones in the Taihu Lake Basin.

Urbanization Zone		Old Urban Area	New Urban Area	Suburbs
$\bar{P}$ * (mm)	Before 1990	1082.75	1056.55	1067.99
	After 1990	1158.05	1149.36	1130.75
$\rho_{P}$	Before 1990	9.2494	9.9483	9.7686
	After 1990	−5.4297	−4.4789	−4.4862
$\rho_{SampEn}$	Before 1990	−0.0013	−0.0006	0.0004
	After 1990	0.0036	0.0024	0.002
*u*		0.00033	0.00021	0.00011

^*^$\bar{P}$ is the average annual precipitation.
